# Supplementary material for: Clay–graphene oxide liquid crystals and their aerogels: synthesis, characterization and properties
Source: R Soc Open Sci. 2019 Feb 20;6(2):181439. doi: 10.1098/rsos.181439 (PMC6408417; doi:10.1098/rsos.181439)
Supplement: POM images of the suspension on a glass slide with 0.1% GO and different concentrations of MMT;POM images of the suspension on a glass slide with 0.3% GO and different concentration of MMT;POM images of the suspension on a glass slide with 0.5% GO and different concentration of MMT [file rsos181439ssupp1.pdf]

Supplementary material

Clay - gaphene oxide Liquid crystals and their aerogels: synthesis, characterization and properties

Sisi Ye, Zhihong Yang*,Jianmei Xu, Zehao Shang, Jing Xie

Faculty of Materials Science and Chemistry, China University of Geosciences, Wuhan, 430074, China

* Corresponding author:

Dr. Zhihong Yang,

Faculty of Materials Science and Chemistry,

China University of Geosciences, Wuhan 430074, P. R. China,

E-mail: yzhh05@126.com, Tel: +86-27-67884814

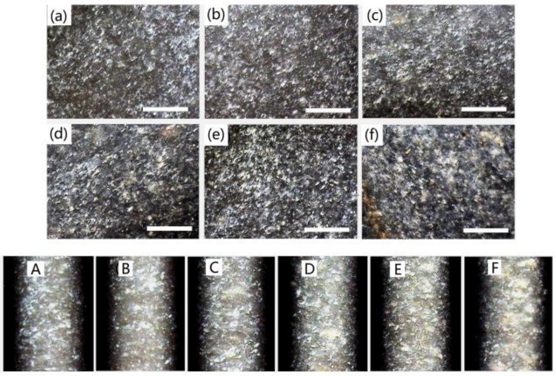

Fig. S1 POM images of the suspension on a glass slide with 0.1% GO and different concentrations of MMT: (a) without MMT, (b) 0.1% MMT，(c) 0.2% MMT, (d) 0.3% MMT, (e) 0.4% MMT, (f) 0.5% MMT. POM images of the corresponding suspensions in a capillary (A-F). Scale bar: 400 μm.

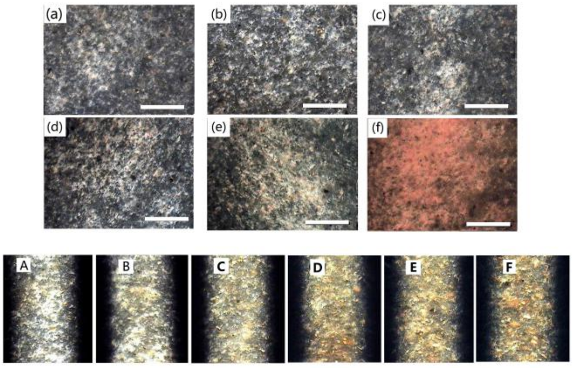

Fig. S2 POM images of the suspension on a glass slide with 0.3% GO and different concentration of MMT: (a) without GO, (b) 0.3% MMT，(c) 0.6% MMT, (d) 0.9% MMT, (e) 1.2% MMT, (f)1.5% MMT. POM images of the corresponding suspensions in a capillary (A-F). Scale bar: 400 μm.

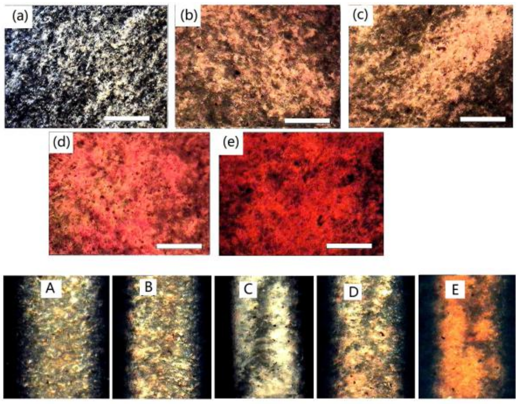

Fig. S3 POM images of the suspension on a glass slide with 0.5% GO and different concentration of MMT: (a) without GO, (b) 0.5% MMT，(c) 1.0% MMT, (d) 1.5% MMT, (e) 2.0% MMT. POM images of the corresponding suspensions in a capillary (A-F). Scale bar: 400 μm.
